# Exploring the pathophysiological consequences of TDP‐43 mislocalization in retinal cells

**DOI:** 10.1002/alz.088965

**Published:** 2025-01-03

**Authors:** Maja Højvang Sørensen, Yan Xu, Virginia M.‐Y. Lee, Sílvia Porta

**Affiliations:** ^1^ Center for Neurodegenerative Disease Research, Department of Pathology and Laboratory Medicine, University of Pennsylvania, Perelman School of Medicine, Philadelphia, PA USA; ^2^ Institute on Aging, University of Pennsylvania, Perelman School of Medicine, Philadelphia, PA USA

## Abstract

**Background:**

Glaucoma is characterized by progressive optic nerve degeneration that results in irreversible blindness, and it can be considered a neurodegenerative disorder of both the eye and the brain. Increasing evidence suggest that glaucoma shares some common neurodegenerative pathways with Frontotemporal Lobar Degeneration (FTLD), Amyotrophic Lateral Sclerosis (ALS), and Alzheimer’s Disease (AD) among others. Interestingly, a recent study revealed the presence of abnormal TAR DNA‐binding protein 43 (TDP‐43) inclusions and aggregates in retinal ganglion cells and other retinal cell types in FTLD‐TDP patients; however, the significance of this pathology and its impact on retinal function and optical nerve integrity is unknown. In these patients, optic nerve degeneration has been linked to the spread of aggregated TDP‐43 beyond the brain. Thus, this study aims to explore the role TDP‐43 mislocalization and aggregation contributes to glaucoma.

**Method:**

The expression of the mutant cytoplasmic human TDP‐43 transgene (hTDP‐43NLSm) in a doxycycline‐regulatable TDP‐43 transgenic mouse line (rNLS8), was induced for 2, 4 and 6 weeks (i.e., Off‐Dox), thus causing a time‐dependent cytoplasmic translocation of TDP‐43 in neurons. rNLS8 mice were euthanized, perfused, and intact eyeballs collected for histological examination. The expression of hTDP‐43 and its phosphorylated form (p409/410) together with cleaved caspase‐3 and p62 was examined by immunohistochemistry in 5 µm paraffin ‐embedded retinal sections.

**Result:**

rNLS8 mice kept Off‐Dox expressed hTDP‐43NLSm in all neuronal layers in the retina, in all the time‐points analyzed. Interestingly, in rNLS8 mice at 6 weeks Off‐Dox, revealed mild phosphorylation of TDP‐43 together with an increase of cleaved caspase‐3 and p62 protein immunoreactivity.

**Conclusion:**

Thus far, this data indicates the activation of apoptotic and autophagy signaling pathways concomitant to the presence of mislocated and phosphorylated TDP‐43. Altogether, this indicates that the rNLS8 transgenic mouse line serves as a good model to evaluate the pathophysiological consequences of TDP‐43 mislocalization in retina cells and its role in optic nerve degeneration.

